# Short version of the right-wing authoritarianism scale for the Brazilian context

**DOI:** 10.1186/s41155-023-00260-4

**Published:** 2023-07-20

**Authors:** Felipe Vilanova, Taciano L. Milfont, Angelo Brandelli Costa

**Affiliations:** 1 Pontifícia Universidade Católica Do Rio Grande Do Sul, Avenida Ipiranga 6681, Building 11, Room 933, Porto Alegre, RS Brazil; 2 University of Waikato, Tauranga, New Zealand

**Keywords:** Authoritarianism, Ideology, Psychometrics, Brazil, Conservatism

## Abstract

Right-Wing Authoritarianism (RWA) is a central predictor of distinct phenomena such as prejudice, voting behavior, corruption, conspiratory beliefs and dietary habits. Given its theoretical and practical relevance, researchers have incorporated RWA measures in large-scale surveys but their length can be an impediment. Although short RWA scales exist, none consider the cultural variability of the RWA structure in non-WEIRD contexts such as Brazil. Here, we report data from five cross-sectional and longitudinal Brazilian samples (*N*_total_ = 2,493) used to develop a short RWA version that considers cross-cultural specificities of the Brazilian context, where an alternative four-factor model was observed. Exploratory and confirmatory factor analyses confirmed a four-factor structure comprising Authoritarianism, Traditionalism, Submission to Authority and Contestation to Authority dimensions. Six-month longitudinal results indicated that Authoritarianism and Traditionalism are more stable than both Submission and Contestation to Authority. Correlations between these dimensions and right-wing political self-categorization were statistically equivalent for the full 34-item RWA scale version and the new 12-item version. Results confirm the psychometric properties of the four-factor, 12-item RWA scale in this cultural context.

**Supplementary Information:**

The online version contains supplementary material available at 10.1186/s41155-023-00260-4.

## Introduction

Political ideologies have been increasingly pointed out as central predictors of distinct socio-psychological phenomena. Daily activities such as media exposure, social media access and even personal relationships are actively selected based on ideological beliefs (Spohr, 2017), demanding research attention for better understandings of its growing influence. Particularly, one ideological dimension indexing individuals’ tendency to conform to social norms has been consistently found in research across distinct societies at least since the 1950s (Adorno et al., 1950; Claessens et al., 2020). This ideological dimension is notable for its relationship with a wide array of phenomena, including voting behavior (Womick, Rothmund, Azevedo, King, and Jost, 2019), prejudice (Sibley et al., 2006), support for torture (Benjamin Jr, 2016), corruption (Vilanova et al., 2022b), belief in conspiracy theories (Wood & Gray, 2019), and even dietary habits (Milfont et al., 2021). Although distinct terms have been used to describe this ideological dimension, such as normativism (Tomkins, 1964), group loyalty (Trompenaars, 1993) and more recently social-cultural right-wing attitudes (Onraet et al., 2013), Right-Wing Authoritarianism (RWA) is one of the most common and accurate definitions (Altemeyer, 1981; for a recent review, see Osborne et al., 2023).

RWA was initially defined as a unidimensional personality trait that emerges from the covariation of three core dimensions (Altemeyer, 1981): “Conventionalism” (i.e., adherence to social norms that are seen as endorsed by established authorities), “Authoritarian Aggression” (i.e., aggressiveness towards various persons, seen as sanctioned by established authorities), and “Authoritarian Submission” (i.e., uncritical submission to authorities seen as established and legitimate). This definition was prominent in the 1980s and 1990s (Altemeyer, 1996) but the conceptualization of RWA as a *unidimensional personality trait* became increasingly questioned by scholars in the 2000s (Duckitt, 2001).

In particular, the personality assumption was empirically challenged by meta-analytical results showing weak correlations between RWA and almost all subdimensions of the Big Five personality traits (Sibley & Duckitt, 2008). Moreover, other work indicated RWA to be more strongly related to social attitudes such as prejudice (Duckitt et al., 2002), support for torture (Duckitt, 2001), and conservative political self-categorization (Jost et al., 2003) than personality traits. In parallel, the unidimensional assumption was questioned when evidence pointed out that a three-dimensional model fit the data better than the original unidimensional model (Funke, 2005; Mavor et al., 2010), indicating that the three core dimensions originally proposed are more adequately measured when assessed separately. Hence, a novel conceptualization of RWA as a multidimensional socio-attitudinal construct was proposed (Duckitt & Sibley, 2017).

Notably, Duckitt et al. (2010) provided an explicit discussion of RWA as a three-dimensional social attitude. They proposed a model of RWA formed by “Authoritarianism”, indexing the endorsement of harsher coercive measures (similar to the dimension of Authoritarian Aggression proposed by Altemeyer, 1981); “Conservatism”, indexing the tendency to submit uncritically to authorities (similar to Authoritarian Submission); and “Traditionalism”, indexing the support for traditional moral values (similar to Conventionalism). This three-dimensional model implies that each dimension has a distinct motivational goal with differential relationships with external variables. To illustrate, whereas the Authoritarianism dimension primarily predicts attitudes towards groups seen as dangerous and threatening, Conservatism primarily predicts attitudes towards groups seen as dissident (Duckitt & Bizumic, 2013). Moreover, Traditionalism seems to be the only dimension predicting religious fundamentalism and ethnicity (i.e., Asian New Zealanders vs European New Zealanders, Duckitt et al., 2010), as well as prejudice towards sexual and gender diversity (Vilanova et al., 2019). Accordingly, this three-dimensional socio-attitudinal model of RWA superseded the original unidimensional personality model.

### Short measures of RWA

Due to its prominent relationships and predictive validity, researchers sought to assess RWA in large-scale social surveys such as the World Values Survey (Haerpfer et al., 2020) or the New Zealand Attitudes and Values Survey (Sibley, 2009). One important impediment is the length of the measures, since available scales are composed of 17 (Feldman, 2003), 30 (Altemeyer, 1996) and even 36 items (Duckitt et al., 2010), being thus unfeasible to incorporate such measures in large-scale surveys with multiple measures. As an alternative, shorter versions of classic instruments have been proposed, such as the 21-item Northern-American version of RWA proposed by Altemeyer (1981), the 15-item Swedish version proposed by Zakrisson (2005), or the 14-item Italian version proposed by Rattazzi et al. (2007). Even though these are successful short versions, they are still based on the older conceptualization of RWA, not considering its current multidimensional socio-attitudinal definition.

Recently, Bizumic and Duckitt (2018) proposed a “very short authoritarianism scale” that is based on the contemporary three-dimensional RWA conceptualization. Their main objective was to propose an instrument that best measures overall RWA instead of each particular dimension. Therefore, they conducted Exploratory Factor Analysis (EFA) and calculated item-total correlations of the 12-item instrument proposed by Duckitt et al. (2010), retaining the 6 items with the highest loadings on a single RWA dimension. They tested distinct models with these 6 items through Confirmatory Factor Analysis (CFA) in samples from Australia, the United Kingdom, and the United States of America and obtained adequate fit indices. For instance, the one-dimension solution presented adequate fit (i.e., CFI and GLI > 0.95; RMSEA < 0.08), and the three-dimension and 1*3 higher-order solutions (one general higher-order RWA dimension and three lower-order dimensions) had even better fit indices in the samples assessed (i.e., CFI and GLI > 0.98; RMSEA < 0.05). The correlations of the short RWA scale with external variables such as nationalism, pro-war attitudes, and ethnocentrism were in the same direction of the long RWA scale and displayed similar magnitudes, providing evidence of its convergent, discriminant and construct validity.

Despite the success of Bizumic and Duckitt (2018) in proposing a short version of the RWA scale, two key limitations regarding the reliability and validity of their scale must be acknowledged. First, it is worth noting that the control of wording effect was explicitly prioritized at the expense of the internal consistency reliability (Bizumic & Duckitt, 2018, p. 132). In fact, RWA was not indexed by the top-loading items regardless of its direction, but rather by the three top-loading pro-trait items and the three top-loading con-trait items, which had somewhat disparate factor loadings ranging from 0.42 to 0.68. The combination of pro-and con-trait items with dissimilar loadings might have reduced the reliability of the instrument, as evidenced by the moderate Cronbach’s α values across the samples assessed, ranging from 0.71 (USA sample, Study 2) to 0.78 (UK sample, Study 2). Second, even though the objective of the study was not to propose a scale that best measures each RWA dimension, two distinct items tapped each of the three dimensions proposed by Duckitt et al. (2010). This could be considered an issue because using less than 3 items to tap each dimension of a multidimensional construct provides rather unstable factor-solutions and reduces its reliability (Bollen, 1989; Costello & Osborne, 2005; Raubenheimer, 2004). Indeed, the inter-item correlations in the study by Bizumic and Duckitt (2018) were small to moderate, ranging from 0.29 (USA sample, Study 2) to 0.34 (USA sample, Study 3).

These limitations might be even more concerning in some contexts, especially non-WEIRD (Western, Educated, Industrialized, Rich and Democratic) countries. In these contexts, the consequences of prioritizing the balance in items directionality might be more severe, since combining positively- and negatively-keyed items in a scale increases not only the time necessary to complete the instrument, but also the frequency of inconsistent responses (Salazar, 2015), especially among individuals with low educational level (Meisenberg & Williams, 2008). Notably, the national contexts of samples assessed by Bizumic and Duckitt (2018) are among the highest average expected years of schooling according to the Human Development Report of the United Nations Development Programme (2020): Australia occupies the 1st position, the UK occupies the 15th, and the USA occupies the 28th; whereas non-WEIRD countries such as Brazil and Egypt occupy the 45th and 94th positions, respectively. Additionally, our research experience indicates that balanced measures are more cognitively taxing for participants in contexts where Likert-type rating scales are not commonly employed in educational settings and broader society. Hence, it is reasonable to expect that the consistency of the answers and the reliability of the measure would be even lower in non-WEIRD countries if the balance in item directionality were prioritized.

Beyond these methodological considerations, cross-cultural differences in the dimensionality of RWA are also important to be considered. Indeed, there is evidence for cultural variability in RWA factor structure, such that the original unidimensional structure proposed by Altemeyer (1981, 1996) had inadequate fit to the data in Argentinian (Etchezahar, 2012), South African (Gray & Durrheim, 2006) and Japanese (Takano et al., 2020) contexts, being thus replaced by multidimensional structures. Even the most recent three-dimensional model proposed by Duckitt et al. (2010) showed cultural variability in the Brazilian context (Vilanova et al., 2018), which is of key relevance for the present study whose main objective is to propose a short version of the RWA scale for the Brazilian population.

### Short RWA in Brazil

Vilanova et al. (2018) provided preliminary evidence that the three-factor structure proposed by Duckitt et al. (2010) was inadequate to the Brazilian context, being thus superseded by a four-factor structure. The original ‘Conservatism’ items clustered into two distinct factors that, although resembling a spurious methodological split between negatively and positively worded items, expressed qualitatively distinct information: whereas the con-trait items expressed contesting authority, being thus named “Contestation to Authority” (e.g., “The more people there are that are prepared to protest against the government, the better it is for society”, Vilanova et al., 2018, p. 1333), the pro-trait items expressed submission to authority, being thus named “Submission to Authority” (e.g., “Our leaders should be obeyed without question”, Vilanova et al., 2018, p. 1334). Later evidence by Vilanova et al. (2020) indicated that the split was not methodological, since these two dimensions were only weakly correlated and had important distinguishing features. To illustrate, their zero-order correlations were consistently small to moderate (ranging from -0.33 to -0.49), and even statistically non-significant when controlling for the influence of the other two RWA factors. Furthermore, their longitudinal stability in a 3-year period was significantly different, such that Contestation to Authority was less stable (Intraclass correlation coefficient = 0.61) than Submission to Authority (ICC = 0.78), Traditionalism (ICC = 0.84) and Authoritarianism (ICC = 0.92).

It is worth noting that Vilanova et al. (2020) provided not only empirical evidence for the four-factor structure, but also theoretical reasons for the split in the original Conservatism factor in the Brazilian context. For instance, content analysis comparing the original items proposed by Duckitt et al. (2010) indicated that many pro- and con-trait items do not refer to the same grammatical objects: whereas the pro-trait items refer to “leaders” (Items 2, 5, and 12), “authority” (Items 4, 5, 10, and 11), and “those who are in charge” (Item 10), con-trait items refer to “authority” (Items 1, 3, 8 and 9), “government” (Items 7 and 8), and “laws” (Item 6). Hence, only “authority” is the common grammatical object across both pro- and con-trait items. These distinct references are important because government and laws (i.e., the focus of the grammatical objects of the original con-trait items) significantly shifted in a short period in Brazil and are arguably more unstable when compared to other countries.

To illustrate, between 2016 and 2023 Brazil had four divergent governments that significantly changed the national legislation. In 2016, Dilma Rousseff was the president, following a center-left orientation and defending policies such as investment in science and technology, popular housing programs, and social security. Rousseff was impeached in August 2016 and her vice-president, Michel Temer, took charge as the new president. Temer followed a center-right orientation, slashing federal funds for science by nearly half, stopping popular housing programs, and proposing a bill-of-law to reform the national social security system. In 2018, Jair Bolsonaro was elected, following a far-right orientation that radicalized Temer’s austere initiatives and significantly changed the structure of the Brazilian state. Notably, Bolsonaro instituted a presidential decree that made it easier to have a gun at home, appointed military personnel to more than 20% of the government ministries for the first time since the Brazilian military dictatorship, and undermined the protection of the Amazon rainforest through a less strict regulation of the economic activities in this area (Greenpeace Brasil, 2022). Lastly, in 2022 Luiz Inácio Lula da Silva, who had been the president of Brazil between 2002 and 2010, was elected again for a third term, endorsing a center-left platform similar to that put forward by Dilma Rousseff. Hence, in the 7-year period between 2016 and 2023 the government and laws in Brazil shifted from a center-left orientation to a center-right orientation, then to a far-right orientation and back to a center-left orientation again (Dantas, 2022).

The political instability was pointed out by Vilanova et al. (2020) as the reason underlying the split of the original pro- and con-trait Conservatism items in that national context. As governments and laws significantly changed in a short period and no drastic revolution happened, right-wing authoritarians in Brazil probably do not consider uncritical submission to government and laws necessary to maintain collective security. Alternatively, they may remain faithful to their leaders even if they are not in the government anymore, such as Bolsonaro after the 2022 elections. Therefore, whereas uncritical submission to the grammatical objects of original pro-trait (i.e., “leaders”, “those who are in charge”) and con-trait Conservatism items (i.e., “government” and “laws”) may be entangled in contexts that are more politically stable, this may not be the case in more politically unstable contexts such as Brazil.

Even though Vilanova et al. (2018) provided a 34-item version of RWA that was cross-culturally adapted to the Brazilian context (see also Cantal, Milfont, Wilson, & Gouveia, 2015), no short versions have been proposed yet. Having a short version is especially important in this country because many funding and time constraints put significant obstacles to research with long measures. For instance, data from UNESCO (2021) indicate that only 1.3% of the Brazilian GDP is spent on research and development, which is considerably smaller than the shares in rich countries such as Germany (2.9%), the USA (2.7%), and Australia (2.2%), providing scarce resources for Brazilian scholars. Moreover, it is legally prohibited to pay individuals for their participation in research conducted in Brazil (Conselho Nacional de Saúde, 2016), and since there are no national online platforms focused on participants recruitment such as MTurk or Prolific, data collection is particularly time-consuming.

The specific objective of this project was thus to propose a short RWA version that considers its multidimensional socio-attitudinal definition, the cross-cultural specificities of the Brazilian context, and overcomes the limitations of the very short authoritarianism scale proposed by Bizumic and Duckitt (2018). Hence, to successfully tap the four culture-specific RWA dimensions with three items each (following methodological guidelines by Bollen, 1989; Costello & Osborne, 2005; Raubenheimer, 2004), we sought to propose a 12-item RWA version. Considering previous research by Vilanova et al., (2018, 2020), we tentatively hypothesized that a four-dimensional structure would be the most suitable for the Brazilian context in comparison to the one- and three-dimensional structures previously proposed by Altemeyer (1981) and Duckitt et al. (2010), respectively (H1). Furthermore, as evidence of criterion validity, we hypothesized that the more participants self-categorized as right-wing in the left–right political spectrum, the higher would be their mean scores in the short versions of the items comprising Authoritarianism, Traditionalism and Submission to Authority factors, but not for Contestation to Authority (H2). Finally, based on the Vilanova et al. (2020) findings we hypothesized that the mean scores in the items comprising the short version of the Contestation to Authority factor would be less stable than the other RWA dimensions in a longitudinal (test–retest) assessment (H3).

## Method

### Participants

Five samples were assessed for the present study, all distinct from those reported by Vilanova et al., (2018, 2020). Sample 1 included 1,110 Brazilians (51.4% male) aged between 18 and 78 years old (*M* = 34.52; *SD* = 12.99) who completed an online survey about social attitudes between October and November 2020. Sample 2 included 999 Brazilians (56.3% male) aged between 18 and 81 years old (*M* = 36.40; *SD* = 15.09) who completed another online survey about personality and social attitudes between April and May 2021. Sample 3 included 90 Brazilians (64.4% male) aged between 18 and 74 years old (*M* = 37.51; *SD* = 16.30) who completed an online survey about authoritarianism between April and May 2018. Sample 4a included 211 Brazilians (55.9% female) aged between 18 and 79 years old (*M* = 32.31; *SD* = 14.72) who completed an online survey about threat perception between June and September 2019 (T1). Of these 211 participants, 140 provided their e-mails for future contact and were invited to answer the RWA scale again in March 2020 (T2). This period was chosen to reassess RWA because Brazil had the most cases and deaths caused by COVID-19 in Latin America and the highest active transmission rate among 48 countries (The Lancet, 2020). Furthermore, two distinct Health Ministers had been removed by former president Jair Bolsonaro, who answered “So What?” when asked by journalists about the rapidly increasing numbers of COVID-19 cases (The Lancet, 2020). Hence, respondents were likely aware of the consequences of the pandemic, and it might have changed their RWA scores given views about authorities’ role in dealing with it. Sample 4b thus included 83 individuals (54.2% female) aged between 18 and 80 years old (*M* = 36.18; *SD* = 15.40) who participated in both T1 and T2 and formed our six-month longitudinal dataset. Although not all participants who provided their e-mail for future contact participated in both waves (40.72% attrition rate), there were no significant differences in age (*t* (136) = -1.67, *p* = 0.10, Cohen’s *d* = 0.28) or gender (χ^2^ (1) = 0.73, *p* = 0.39, Cramer’s *V* = 0.07) between those who participated only in T1 and those who participated in both T1 and T2.

Before answering the instruments, participants expressed their consent by providing their agreement to an Informed Consent Form. Anonymity was granted and only researchers had access to the data. All samples were recruited through convenience sampling and the study design was approved by the ethics committee of the first author’s university.

### Measures

Participants in all samples completed the full version of the RWA scale proposed by Duckitt et al. (2010), which was adapted to the Brazilian context by Vilanova et al. (2018). As explained in the Introduction section, the RWA measure in Brazil comprises 34 items split into four factors: Authoritarianism (AT), Traditionalism (TR), Submission to Authority (SA), and Contestation to Authority (CA). Responses are given on a 5-point agreement scale ranging from 1 (strongly disagree) to 5 (strongly agree).

As in previous independent studies (Vilanova et al., 2018, 2022a, 2022b), political self-categorization was assessed. Participants in Samples 1, 2, 3 and 4a indicated their political self-categorization as “left”, “center-left”, “center”, “center-right”, or “right”, with “none of the aforementioned” also included as a response option. The “none of the aforementioned” category was not considered for the Pearson correlational analyses described in the following section (i.e., *n* = 476 in Sample 1, *n* = 448 in Sample 2, *n* = 9 in Sample 3, *n* = 28 in Sample 4a), and the other categories were coded such that a higher score indicated a greater right-wing political orientation (i.e., left = 1; center-left = 2; center = 3; center-right = 4; right = 5).

### Data analysis

#### Assessing the validity of the short RWA scale

Sample 1 was used to obtain the items of the short version of the RWA scale through EFA and then compare its suggested structure with one-, three- and four-dimensional models through CFA. Samples 2, 3, 4a and 4b were subsequently used to replicate the statistical comparison of the models. Hence, the first step of our analytical procedure was to conduct a parallel analysis using Monte Carlo simulation in Sample 1 to verify how many factors could be reliably extracted. Factors that met the Kaiser-Guttman criterion (i.e., Eigenvalue > 1) and displayed higher Eigenvalues than the generated by the simulations were retained. Next, an EFA using principal axis factoring and Oblimin rotation was conducted in Sample 1 to assess the 3 items with the highest factor loadings in each dimension. If two or more items had the same loading, the one with higher item-total correlation for the corresponding dimension was retained.

After the three items with higher factor loadings were extracted, the model obtained by the EFA in Sample 1 was tested through CFA in this and the other samples. Values of χ^2^/df < 2, RMSEA ≤ 0.06, CFI and TLI ≥ 0.95 were deemed adequate (Bollen & Long, 1993; Hu & Bentler, 1999). The χ^2^ difference test was then conducted to test if the model obtained through EFA had a significantly better fit than the original three-factor structure proposed by Duckitt et al. (2010), as well as the one-factor structure proposed by Altemeyer (1981).

To assess the criterion validity of the instrument, Pearson correlations between the mean scores of the RWA dimensions and political self-categorization were calculated. These correlations were then compared across the long and short versions of the RWA scale to verify if their relationships with this external variable were equivalent. The 95% confidence intervals of the correlations between self-categorization and the long and short RWA versions were calculated, and if the confidence intervals overlapped, they were considered equivalent. Furthermore, we tested the significance of the difference between the correlations of the RWA dimensions and political self-categorization across the long and short versions using the *z*-test calculator proposed by Soper (2023).

### Assessing the reliability of the short RWA scale

To assess the reliability of the instrument, two distinct analyses were conducted. First, Cronbach’s alpha and McDonald’s omega of each dimension were calculated for all samples. Second, the Intraclass Correlation Coefficients (ICCs) of the mean dimensional scores were calculated across T1 and T2 of Sample 4b to assess the test–retest longitudinal stability of the measure, based on a single measurement, absolute-agreement two-way mixed-effects model (Koo & Li, 2016).

## Results

As Sample 1 was used to obtain the items of the short version of the RWA scale, the EFA and CFA results of this sample will be first described. Then, results will be compared across the other four samples.

### Validity evidence of the short RWA scale

EFA and parallel analysis in Sample 1 indicated that four factors could be reliably extracted. Only four factors extracted by EFA had Eigenvalues greater than 1 (i.e., 10.12, 2.78, 1.69 and 1.53) and they were higher than Eigenvalues obtained from data simulation (i.e., 0.38, 0.31, 0.28 and 0.25), so a four-factor solution was extracted. Table 1 shows the EFA results. As can be seen, at least three items with very high factor loadings (> 0.70) were observed in each dimension. The only factor in which two items presented the same loading was Authoritarianism (Items 1 and 6) so the item-total correlations for this factor were compared to decide which of these items to retain. Item 6 presented a higher item-total correlation (0.80) than Item 1 (0.75), so the former was retained.

**Table 1:** Rotated Factor Loadings Obtained Through Exploratory Factor Analysis in Sample 1

Item	AT	SA	CA	TR
**The way things are going in this country, it’s going to take a lot of “strong medicine” to straighten out the troublemakers, criminals, and perverts**	**.75**	.06	.05	.02
2	.74	.05	.05	-.02
**Being kind to criminals will only encourage them to take advantage of your weakness, so it’s best to use a firm, tough hand when dealing with them**	**.76**	.03	.05	.01
4	-.55	.06	.00	.09
**The facts on crime and the recent public disorders show we have to crack down harder on troublemakers, if we are going preserve law and order**	**.78**	.09	-.01	.01
**What our country really needs is a tough, harsh dose of law and order**	**.75**	.09	.00	.06
7	.64	.15	.15	-.04
8	-.58	.15	.19	-.06
9	-.59	.23	.24	-.04
10	-.58	.09	.13	-.02
11	-.65	.03	.08	-.05
**The more people there are that are prepared to challenge the government, the better it is for society**	.07	-.01	**.73**	-.05
13	-.02	-.02	.69	-.06
14	.01	-.10	.68	.00
15	.09	-.04	.63	.03
**Students at high schools and at university must be encouraged to challenge, criticize, and confront authorities**	-.08	-.05	**.73**	.02
**It’s great that many young people today are prepared to defy authority**	-.06	-.08	**.74**	.01
**Everyone should have their own sexual preferences, even if it makes them different from everyone else**	-.04	.00	-.07	**-.76**
**There is nothing wrong with premarital sexual intercourse**	.04	-.09	-.06	**-.77**
20	-.05	-.04	.02	-.65
**Everyone should have their own lifestyle, even if it makes them different from everyone else**	.04	.06	.02	**-.74**
22	.00	.07	-.04	-.59
23	-.07	-.05	.19	-.50
24	.12	.25	-.05	.47
25	-.02	.01	.24	-.55
26	-.08	.02	.20	-.24
27	-.04	.66	.02	.08
28	-.04	.70	-.06	.01
**Our country will be great if we obey our leaders**	-.02	**.76**	-.06	.05
**The real key to the “good life” is respect for authority**	.02	**.78**	-.03	-.01
**What our country needs most is discipline, with everyone following our leaders in unity**	.09	**.76**	-.02	-.03
32	-.07	.58	.00	.10
33	.14	.66	.00	.01
34	.06	.65	-.12	.01

Note: Top-three factor loadings in bold; Full items can be found in Supplementary Material A; *AT* Authoritarianism, *CA* Contestation to Authority, *TR* Traditionalism, *SA* Submission to Authority

CFAs in Sample 1 indicated that the four-factor structure indicated by EFA is the best fitting for the Brazilian context. The fit indices of the four-factor structure were good (χ^2^/df = 3.34; CFI = 0.97; TLI = 0.96; RMSEA = 0.046, 90% C.I. [0.038, 0.054]), whereas the same items organized according to the original three-factor structure proposed by Duckitt et al. (2010) had inadequate fit to the data (χ^2^/df = 15.30; CFI = 0.83; TLI = 0.78; RMSEA = 0.114, 90% C.I. [0.107, 0.121]), and it was a significantly poorer fitting model than the four-factor model (χ^2^_difference_ (3) = 72.97, *p* < 0.001). Similarly, the one-factor structure proposed by Altemeyer (1981) also had inadequate fit to the data (χ^2^/df = 28.49; CFI = 0.66; TLI = 0.58; RMSEA = 0.157, 90% C.I. [0.151, 0.164]) and was a significantly poorer fitting model than the four-factor model (χ^2^_difference_ (3) = 366.58, *p* < 0.001).

As evidence of criterion validity, Pearson correlations indicated that the more the participants categorized themselves as right-wing politically, the higher were their scores in the short versions of the factors capturing Authoritarianism (*r* (542) = 0.67, *p* < 0.001, 95% CI [0.62, 0.71]), Submission to Authority (*r* (542) = 0.55, *p* < 0.001, 95% CI [0.49, 0.61]) and Traditionalism (*r* (542) = 0.49, *p* < 0.001, 95% CI [0.42, 0.55]), whereas the opposite pattern was found for Contestation to Authority (*r* (542) = -0.49, *p* < 0.001, 95% CI [-0.55, -0.42]). When using the long version of the instrument, similar correlations were found between political self-categorization and the four factors: Authoritarianism (*r* (542) = 0.70, *p* < 0.001, 95% CI [0.66, 0.74]), Submission to Authority (*r* (542) = 0.56, *p* < 0.001, 95% CI [0.50, 0.62]), Traditionalism (*r* (542) = 0.60, *p* < 0.001, 95% CI [0.55, 0.65]) and Contestation to Authority (*r* (542) = -0.49, *p* < 0.001, 95% CI [-0.55, -0.42]). Notably, the confidence intervals of all correlations between political self-categorization and the short- and long- versions of the RWA scale overlapped, indicating that these correlations are equivalent. Furthermore, *z*-tests indicated no significant difference across the short and long versions of the instrument considering the correlations between political self-categorization and Authoritarianism (*z* = -0.93, *p* = 0.35), Submission to Authority (*z* = -0.24, *p* = 0.81), and Contestation to Authority (*z* = 0, *p* = 1), except for Traditionalism (*z* = -2.58, *p* = 0.01) which supports the marginal overlap reported above for the correlation coefficients (i.e., short form: 0.49, 95% CI [0.42, 0.55], long form: 0.60, 95% CI [0.55, 0.65]). 

Item-total correlations, CFAs and correlations between RWA factors and political self-categorization were then calculated to assess the validity of the short RWA scale in Samples 2, 3, 4a and 4b. As shown in Table 2, item-total correlations were adequate across all samples, ranging from 0.52 (Item 12 in Sample 2) to 0.91 (Item 18 in Sample 4b). Similarly, Table 3 indicates that fit indices of the four-factor structure were adequate in all samples and significantly better than the original three- and one-dimensional models in most samples. Finally, Supplementary Material B indicates the similarity between the short and long versions of the RWA scale, as illustrated by the overlap of the confidence intervals of all Pearson correlations with political self-categorization, the overall *z*-test results, and the overlap of the meta-analyzed correlation coefficients across all samples. To illustrate, the stronger correlation with right-wing political orientation was with the Authoritarianism dimension in Sample 3, which was comparable across the long (*r* (79) = 0.80, *p* < 0.001, 95% CI [0.70, 0.87]) and short (*r* (79) = 0.84, p < 0.001, 95% CI [0.76, 0.89]) versions. Moreover, the meta-analyzed correlation coefficient between right-wing political orientation and Authoritarianism (shown in Supplementary Material B) was 0.95 (95% CI [0.77, 1.14]) and 0.98 (95% CI [0.78, 1.18]) for the long and short versions, respectively. The validity of the short RWA scale was thus supported, and these findings provide support to Hypotheses 1 and 2.

**Table 2:** Item-Total Correlations for each Dimension in Samples 2, 3, 4a and 4b

(Sample 2; Sample 3; Sample 4a; Sample 4b)				
Item #	AT	CA	TR	SA
3	.70; .85; .78; .85			
5	.83; .87; .90; .83			
6	.75; .85; .85; .88			
12		.52; .68; .63; .67		
16		.81; .70; .85; .81		
17		.85; .56; .91; .90		
18			.74; .85; .79; .91	
19			.72; .68; .62; .73	
21			.76; .74; .77; .70	
29				.76; .76; .75; .79
30				.68; .58; .74; .86
31				.70; .77; .78; .88

Note: Full items can be found in Supplementary Material A; *AT* Authoritarianism, *CA* Contestation to Authority, *TR* Traditionalism, *SA* = Submission to Authority

**Table 3:** Model Fit for Four-Factor, Three-Factor and One-Factor Models in Samples 2, 3, 4a and 4b

Sample	Model	χ^2^/*df*	CFI	TLI	RMSEA	χ^2^_difference_ test		
						χ^2^_difference_	*df*_difference_	*p*
Sample 2 (*n* = 990)	Four-Factor (AT, TR, SA, CA)	3.50	.96	.94	.05	-	*-*	*-*
	Three-Factor proposed by Duckitt et al., (2010; AT, TR, SA + CA)	17.58	.72	.63	.13	253.93	3	< .001
	One-Factor Proposed by Altemeyer (1981)	31.27	.45	.33	.18	414.91	3	< .001
Sample 3 (*n* = 90)	Four-Factor (AT, TR, SA, CA)	1.09	.99	.98	.03	-	-	-
	Three-Factor proposed by Duckitt et al., (2010; AT, TR, SA + CA)	1.62	.89	.86	.08	19.65	3	< .001
	One-Factor Proposed by Altemeyer (1981)	1.87	.84	.81	.10	15.93	3	.001
Sample 4a (*n* = 211)	Four-Factor (AT, TR, SA, CA)	1.28	.97	.96	.04	-	-	-
	Three-Factor proposed by Duckitt et al., (2010; AT, TR, SA + CA)	3.36	.76	.69	.11	4.96	3	.17
	One-Factor Proposed by Altemeyer (1981)	4.36	.64	.56	.13	4.00	3	.26
Sample 4b	Four-Factor (AT, TR, SA, CA)	.88	1	1	0	-	-	-
(*n* = 83)	Three-Factor proposed by Duckitt et al., (2010; AT, TR, SA + CA)	1.43	.93	.91	.07	3.39	3	.34
	One-Factor Proposed by Altemeyer (1981)	1.75	.87	.83	.10	2.67	3	.45

Note: *AT* Authoritarianism, *CA* Contestation to Authority, *TR* Traditionalism, *SA* Submission to Authority

### Reliability Evidence of the Short RWA Scale

The Cronbach’s α and McDonald’s ω of the short RWA dimensions provide indication of their reliability. The α and ω values in Sample 1 were adequate for Authoritarianism (0.86 and 0.86, respectively), Contestation to Authority (0.81, 0.83), Submission to Authority (0.85, 0.85), and Traditionalism (0.79, 0.78). Results reported in Table 4 confirmed the reliability indices were consistent across all other samples, with α values ranging from 0.73 (Contestation to Authority in Sample 3) to 0.91 (Authoritarianism in Sample 3), and ω values ranging from 0.79 (Submission to Authority in Sample 4a) to 0.91 (Authoritarianism in Sample 3).

**Table 4:** Cronbach’s Alpha, McDonald’s Omega and Intraclass Correlation Coefficients of Short RWA Dimensions

Sample	RWA Dimension	Cronbach’s α	McDonald’s ω	ICC
Sample 2 (*n* = 990)	AT	.83	.83	-
	CA	.79	.81	-
	SA	.82	.82	-
	TR	.79	.79	-
Sample 3 (*n* = 90)	AT	.91	.91	-
	CA	.73	.73	-
	SA	.78	.77	-
	TR	.82	.82	-
Sample 4a (*n* = 211)	AT	.90	.90	-
	CA	.85	.86	-
	SA	.83	.79	-
	TR	.80	.83	-
Sample 4b (*n* = 83)	AT	.90	.90	.92
	CA	.85	.89	.75
	SA	.90	.90	.73
	TR	.83	.83	.93

Note: *AT* Authoritarianism, *CA* Contestation to Authority, *SA* Submission to Authority, *TR* Traditionalism. *ICC* Intraclass Correlation Coefficient considering T1 (June–September 2019) and T2 (March 2020) of Sample 4b

Finally, the six-month test–retest of the measure across T1 and T2 of Sample 4b indicated dissimilar longitudinal stability of the factors. Rejecting Hypothesis 3, Contestation to Authority was not the most unstable factor since its ICC (0.75) was greater than that of Submission to Authority (0.73). Nevertheless, the ICCs for Traditionalism (0.93) and Authoritarianism (0.92) were according to our expectations.

## Discussion

Right-Wing Authoritarianism (RWA) is a multidimensional socio-attitudinal construct predictive of distinct socio-psychological phenomena. There are many RWA measures available but psychometrically sound short scales that can be more easily applied in resource-scarce contexts are missing. The objective of this study was to propose a short version of the RWA scale that considers the specificities of the construct in Brazil and has a sufficient number of items that affords adequate measurement of the contemporary RWA model. Evidence for its validity and reliability was found across four main samples and a six-month test–retest sample (*N* > 2,400). As hypothesized, the four-factor structure had the best fitting model, and the more participants self-categorized themselves as right-wing in the left–right political spectrum, the higher their mean scores on the Authoritarianism, Traditionalism, and Submission to Authority factors. The opposite pattern was found for the Contestation to Authority factor, which unexpectedly was not the most unstable factor in the period before (June–September 2019) and during the COVID-19 pandemic (March 2020).

Contrary to the focus of Bizumic and Duckitt (2018) in proposing a measure that best captures overall RWA, our goal was to identify the three psychometrically stronger items to create the best measure of each RWA dimension for the Brazilian context. Indeed, the values of Cronbach’s α and McDonald’s ω, as well as the inter-item correlations and factor loadings tended to be higher in our samples. For instance, whereas in the samples of Bizumic and Duckitt (2018) mean α and ω values were 0.75, in our samples they were 0.83 and 0.84, respectively. Similarly, whereas the mean item-total correlation in the development sample of Bizumic and Duckitt (2018) was 0.54 (only the item-total correlation for this sample was provided), in our samples it was 0.77. Finally, whereas the mean factor loading of the items retained in their very short authoritarianism scale was 0.58, in our sample it was 0.76. In general, there is thus an indication that the items retained in our measure are more strongly related to the constructs the items tap and consequently more likely to provide a higher consistency rate in the answers—although it is worth noting that the measure we proposed is composed of more items and thus reliability coefficients tend to be higher.

Similar to Vilanova et al. (2020), the only factor structure that had adequate fit indices across all Brazilian samples was the four-dimensional model, indicating that the split between the original pro- and con-trait Conservatism items is not driven by methodological aspects but rather by qualitative distinctions between the content of the items in this cultural context. The fit indices of the four-dimensional model were significantly better than those of the three-dimensional model in Samples 1, 2 and 3, and even though they did not significantly differ in Samples 4a and 4b, this is probably a sample-specific issue, since they also significantly differed in the four samples assessed by Vilanova et al. (2020). Indeed, the mean correlation between the short versions of Submission to Authority and Contestation to Authority in our samples was moderate at best (-0.39), indicating that they do not compose one single construct.

Despite the different procedures, it is worth noting that four out of the six items retained in the very short authoritarianism scale proposed by Bizumic and Duckitt (2018) were also retained in our version. Hence, the issues indexed by items 5 (i.e., endorsement of harsher coercive measures), 17 (i.e., young defiance to authority), 19 (i.e., premarital sex) and 31 (i.e., national need for discipline and unity) seem to be central for RWA even in contexts with clearly different cultural features and varying levels of political stability. This might explain the similarities between recent right-wing governments in the world such as those led by Jair Bolsonaro in Brazil, Donald Trump in the USA and Viktor Orbán in Hungary, all of whom addressed the same topics even if portraying them in seemingly context-relevant ways. For instance, whereas Trump frequently mentioned the “threat” of Latin and Chinese immigration to the USA (De Jonge, 2016)—often associating it with an increase in crime rates and the necessity for harsher coercive measures—a very similar discourse was used by Orbán about Muslims immigrating to Hungary (Schultheis, 2018) as well as by Bolsonaro in relation to Venezuelans immigrating to Brazil (Alegretti, 2019). Furthermore, the call for national unity based on allegedly Christian values has also been often addressed, characterizing some of the main campaign strategies in these three countries (Green et al., 2006). The fact that items addressing these issues were retained in samples from distinct countries assessing RWA, combined with the fact these three politicians were elected based on their views about these issues, shows how ideologically significant such social issues might be and provides indication these RWA items are likely culturally equivalent as well as culture-differentiating indicators.

Another important aspect that should be considered is the different stability of RWA factors. Following the results of Vilanova et al. (2020) we had hypothesized that Contestation to Authority would be the less stable factor, but this hypothesis was rejected. Even though the Submission to Authority factor was the most unstable, its ICC (0.73) was similar to that of Contestation to Authority (0.75), while lower than those of Traditionalism (0.93) and Authoritarianism (0.92). Hence, even after the COVID-19 outbreak that killed hundreds of thousands of people, significantly affected political discussions, and even the possibility of daily mobility through lockdowns, Traditionalism and Authoritarianism levels remained stable. These findings thus indicate that the discussions regarding the stability or instability of RWA (e.g., Bonanno & Jost, 2006; Echebarria-Echabe & Fernández-Guede, 2006; Ludeke & Krueger, 2013) should be framed concerning its particular dimensions instead of overall RWA. In combination, these findings suggest that both Traditionalism and Authoritarianism are more trait-like dimensions of RWA, while dimensions related to contesting/following authority are more malleable.

Notwithstanding the many contributions, our study has important limitations that should be considered. First, Samples 3 and 4b were small and did not follow the recommended 1:10 item participant ratio for factor analysis (Costello & Osborne, 2005). Furthermore, the small sample sizes for Samples 4a and 4b should also induce caution in the reliability of the estimates of longitudinal stability, and on the evaluation of the seeming differences or similarities among the ICCs computed on such relatively small samples. Notably, not only Samples 4a and 4b, but also Sample 3 is likely underpowered to replicate the statistical comparison of the models due to its small sample size, possibly inflating false positive and negative rates (Gelman & Carlin, 2014; Gelman et al., 2020). Notwithstanding these points, the fit indices of the factor structures as well as the correlations with political orientation observed in these samples were similar to those observed in other samples, indicating the validity of our findings. Second, only the relationship with one external variable (political self-categorization) was assessed as evidence of criterion validity. Future studies should thus seek to assess the relationship between the short RWA and other variables that are traditionally related to it such as prejudice, support for torture, ethnocentrism, and nationalism (Duckitt et al., 2010). Third, the short RWA scale is still relatively large, being thus unfeasible to incorporate all its 12 items in certain large-scale social surveys. To overcome this limitation, authors can select the items on an ad-hoc basis, using only the items that tap the dimensions of interest for their research (e.g., Almeida-Segundo, 2019)—although we recommend selecting items tapping all three RWA factors. Fourth, even though the analytical procedure provided items with adequate validity and reliability evidence, the lack of con-trait items makes the measure more prone to acquiescence bias. This is a negative effect of our methodological decisions, but we considered that the better psychometric properties outweigh the risk of acquiescence bias. Fifth, we did not test the short version of the scale in a separate sample. All analyses were carried out in samples that answered the long version of RWA, so future studies should now use the short version and assess its psychometric properties.

## Conclusion

This is the first proposition of a short version of the RWA scale in Brazilian-Portuguese according to the most recent multidimensional socio-attitudinal definition of RWA. The cultural variability of the RWA factor structure in the Brazilian context has been taken into account and a 12-item version of the instrument was successfully proposed (available at https://osf.io/admxh/?view_only=7e10fab8061f493da018b16221325b56). Therefore, Brazilian researchers can now use a short sound instrument that might help them overcome the local time and funding constraints of the scientific enterprise.

## Supplementary Information

**Additional file 1.**

## Data Availability

All data and materials are available at https://osf.io/admxh/.

## Abbreviations

RWA: Right-Wing Authoritarianism
WEIRD: Western, Educated, Industrialized, Rich, Democratic
CFA: Confirmatory Factor Analysis
EFA: Exploratory Factor Analysis
CFI: Comparative Fit Index
TLI: Tucker-Lewis Index
RMSEA: Root Mean Square Error of Approximation
UK: United Kingdom
USA: United States of America
UNESCO: United Nations Educational, Scientific, and Cultural Organization
GDP: Gross Domestic Product
M: Mean
SD: Standard Deviation
AT: Authoritarianism
TR: Traditionalism
SA: Submission to Authority
CA: Contestation to Authority
ICC: Intraclass Correlation Coefficient
C.I.: Confidence Interval
