# Supplementary material for: Short version of the right-wing authoritarianism scale for the Brazilian context
Source: Psicol Reflex Crit. 2023 Jul 20;36:17. doi: 10.1186/s41155-023-00260-4 (PMC10359236; doi:10.1186/s41155-023-00260-4)
Supplement: Supplementary file 1 — Additional file 1. [file 41155_2023_260_MOESM1_ESM.docx]

**Supplementary Material A – Full items description**

| Item # |  |
| --- | --- |
| 1 | Do jeito que as coisas estão indo nesse país, serão necessárias medidas severas para endireitar os meliantes, os criminosos e os pervertidos. |
| 2 | A situação do nosso país está ficando tão séria que ações firmes seriam justificadas se eliminassem os desordeiros e nos levassem de volta ao nosso verdadeiro caminho. |
| 3 | Ser gentil com criminosos só os encoraja a tirar proveito de sua fraqueza, sendo melhor agir de maneira firme e dura com eles. |
| 4 | A pena de morte é bárbara e nunca justificável. |
| 5 | Os crimes e as desordens públicas recentes mostram que se quisermos preservar a lei e a ordem, devemos agir de forma mais dura com os desordeiros. |
| 6 | O que o nosso país realmente precisa é uma dose forte e dura de lei e ordem. |
| 7 | Nós deveríamos esmagar todos os elementos negativos que estão causando problemas na nossa sociedade. |
| 8 | Nossas prisões são um desastre. Ao invés de tanta punição, os que estão em conflito com a lei merecem um cuidado muito melhor. |
| 9 | Nós precisamos de maior tolerância e mais leniência no tratamento de infratores. |
| 10 | As pessoas que dizem que nossas leis deveriam ser aplicadas de maneira mais rigorosa e severa estão erradas. |
| 11 | Nossa sociedade NÃO precisa de um governo mais duro e leis mais rigorosas. |
| 12 | Quanto maior o número de pessoas preparadas para desafiar o governo, melhor para a sociedade. |
| 13 | Quanto maior o número de pessoas preparadas para protestar contra o governo, melhor para a sociedade. |
| 14 | Quanto maior o número de pessoas preparadas para criticar as autoridades, melhor para a sociedade. |
| 15 | As pessoas devem estar prontas para desafiar leis com as quais elas não concordam. |
| 16 | Estudantes de colégios e universidades devem ser encorajados a desafiar, criticar e confrontar autoridades. |
| 17 | É ótimo que atualmente muitos jovens estejam preparados para desafiar a autoridade. |
| 18 | As pessoas deveriam ter as suas próprias preferências sexuais, mesmo se isso torná-las diferentes do resto da sociedade. |
| 19 | Não há nada de errado com sexo antes do casamento. |
| 20 | Não tem absolutamente nada errado com praias de nudismo. |
| 21 | As pessoas deveriam ter os seus próprios estilos de vida mesmo se isso torná-las diferentes do resto da sociedade. |
| 22 | As pessoas deveriam ter as suas próprias crenças religiosas mesmo se isso torná-las diferentes do resto da sociedade. |
| 23 | As pessoas deveriam prestar menos atenção à Bíblia e às outras formas antiquadas de orientação religiosa, e desenvolver seu próprio padrão do que é moral e imoral. |
| 24 | As leis de Deus sobre aborto, pornografia e casamento devem ser seguidas à risca antes que seja tarde demais. |
| 25 | Ninguém deveria se restringir ao que é moralmente correto. Ao invés disso, as pessoas deveriam se libertar e experimentar várias ideias e experiências diferentes. |
| 26 | Tem muita coisa de errado com os valores, os costumes e a moralidade tradicionais. |
| 27 | O segredo para uma boa vida é a obediência àqueles que estão no controle. |
| 28 | As autoridades devem ser obedecidas porque elas estão na melhor posição para saber o que é bom para o país. |
| 29 | Nosso país será melhor se obedecermos nossos líderes. |
| 30 | O segredo para uma boa vida é o respeito pela autoridade. |
| 31 | O que nosso país mais precisa é disciplina, com todos seguindo nossos líderes. |
| 32 | Nossos líderes deveriam ser obedecidos sem questionamento. |
| 33 | As virtudes mais importantes que as crianças devem aprender são obediência e respeito à autoridade. |
| 34 | Nosso país será melhor se mostrarmos respeito à autoridade. |

**Supplementary Material B –**

**Table S1.** Pearson Correlations and 95% Confidence Interval Between Political Self-Categorization, Short- and Long- RWA Dimensions

| Sample | Variables | 1 | 2 | 3 | 4 | 5 | 6 | 7 | 8 | *Z*-test *(p*-value*)* |
| --- | --- | --- | --- | --- | --- | --- | --- | --- | --- | --- |
| Sample 2 | 1.Political Self-Categorization | - |  |  |  |  |  |  |  | - |
|  | 2. Short AT | .62 [.56, .67]** | - |  |  |  |  |  |  | 0 (1) |
|  | 3. Long AT | .62 [.57, .67]** | .91 [.89, .92]** | - |  |  |  |  |  | - |
|  | 4. Short CA | -.47 [-.53, -.40]** | -.32 [-.39, -.24]** | -.30 [-.38, -.23]** | - |  |  |  |  | .84 (.40) |
|  | 5. Long CA | -.44 [-.50, -.37]** | -.29 [-.37, -.21]** | -.28 [-.35, -.20]** | .94 [.93, .95]** | - |  |  |  | - |
|  | 6. Short SA | .40 [.33, .47]** | .42 [.35, .49]** | .36 [.29, .43]** | -.32 [-.39, -.24]** | -.35 [-.43, -.28]** | - |  |  | -1.36 (.18) |
|  | 7. Long SA | .45 [.38, .51]** | .48 [.41, .54]** | .43 [.36, .50]** | -.35 [-.42, -.27]** | -.39 [-.46, -.31]** | .93 [.91, .94]** | - |  | - |
|  | 8. Short TR | .48 [.41, .54]** | .34 [.27, .41]** | .34 [.26, .41]** | -.35 [-.42, -.27]** | -.38 [-.45, -.31]** | .44 [.37, .51]** | .49 [.42, .55]** | - | -2.77 (.01) |
|  | 9. Long TR | .57 [.52, .63]** | .46 [.39, .52]** | .45 [.38, .51]** | -.48 [-.54, -.42]** | -.51 [-.57, -.44]** | .47 [.40, .53]** | .52 [.46, .58]** | .86 [.84, .88]** | - |
| Sample 3 | 1.Political Self-Categorization | - |  |  |  |  |  |  |  | - |
|  | 2. Short AT | .84 [.76, .89]** | - |  |  |  |  |  |  | .80 (.42) |
|  | 3. Long AT | .80 [.70, .87]** | .95 [.92, .97]** | - |  |  |  |  |  | - |
|  | 4. Short CA | -.60 [-.73, -.45]** | -.63 [-.74, -.47]** | -.58 [-.70, -.41]** | - |  |  |  |  | .10 (.92) |
|  | 5. Long CA | -.59 [-.72, -.43]** | -.64 [-.75, -.49]** | -.59 [-.72, -.43]** | .94 [.91, .96]** | - |  |  |  | - |
|  | 6. Short SA | .52 [.34, .66]** | .64 [.49, .75]** | .63 [.48, .75]** | -.37 [-.54, -.16]** | -.35 [-.52, -.14]* | - |  |  | -1.20 (.23) |
|  | 7. Long SA | .64 [.49, .75]** | .73 [.61, .82]** | .73 [.60, .81]** | -.44 [-.60, -.24]** | -.44 [-.60, -.25]** | .94 [.91, 96]** | - |  | - |
|  | 8. Short TR | .48 [.29, .63]** | .49 [.30, .64]** | .53 [.35, .67]** | -.32 [-.50, -.11]* | -.23[-.43, -.16]* | .56 [.39, .69]** | .56 [.39, .70]** | - | -.92 (.36) |
|  | 9. Long TR | .58 [.41, .71]** | .63 [.47, .74]** | .64 [.49, .75]** | -.51 [-.65, -.32]** | -.47 [-.62, -.28]** | .64 [.49, .76]** | .67 [.53, .78]** | .87 [.81, .92]** | - |
| Sample 4a | 1.Political Self-Categorization | - |  |  |  |  |  |  |  | - |
|  | 2. Short AT | .79 [.72, .84]** | - |  |  |  |  |  |  | -.28 (.78) |
|  | 3. Long AT | .80 [.74, .84]** | .94 [.92, .96]** | - |  |  |  |  |  | - |
|  | 4. Short CA | -.57 [-.66, -.46]** | -.58 [-.67, -.47]** | -.58 [-.67, -.48]** | - |  |  |  |  | -.31 (.76) |
|  | 5. Long CA | -.59 [-.68, -.49]** | -.59 [-.67, -.48]** | -.60 [-.68, -.49]** | .96 [.95, .97]** | - |  |  |  | - |
|  | 6. Short SA | .66 [.56, .73]** | .68 [.59, .75]** | .66 [.57, .74]** | -.48 [-.59, -.36]** | -.52 [-.62, -.41]** | - |  |  | -.76 (.45) |
|  | 7. Long SA | .70 [.61, .76]** | .72 [.64, .78]** | .70 [.62, .77]** | -.54 [-.64, -.43]** | -.57 [-.66, -.47]** | .96 [.94, .97]** | - |  | - |
|  | 8. Short TR | .56 [.45, .65]** | .56 [.45, .65]** | .52 [.41, .62]** | -.50 [-.60, -.38]** | -.53 [-.63, -.42]** | .52 [.41, .62]** | .53 [.42, .63]** | - | -1.10 (.27) |
|  | 9. Long TR | .63 [.54, .71]** | .61 [.51, .69]** | .62 [.52, .70]** | -.57 [-.66, -.46]** | -.60 [-.69, -.50]** | .60 [.50, .69]** | .63 [.53, .71]** | .85 [.81, .89]** | - |

Note: * *p* < .05; ** *p* < .001; Short = Short version of the dimensions; Long = Long version of the dimensions; AT = Authoritarianism; CA = Contestation to Authority; TR = Traditionalism; SA = Submission to Authority; *Z*-test refers to the *z* value testing the difference between the correlation coefficients of political self-categorization and the short and long versions of the dimensions.

**Figure 1.** Forest plot summarizing the meta-analyzed correlation coefficients between political self-categorization and the short and long versions of RWA dimensions across Samples 2, 3 and 4a


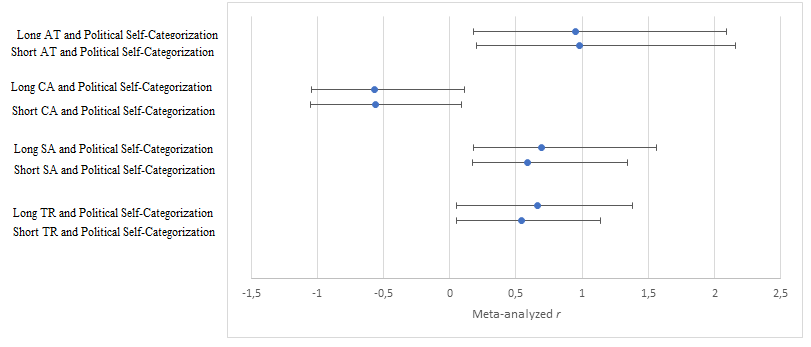


Note: The meta-analysis on the correlation coefficients was conducted using the Hunter-Schmidt estimator for a Random-Effects model based on the Fisher’s *z* transformed correlation coefficient; Short = Short version of the dimensions; Long = Long version of the dimensions; AT = Authoritarianism; CA = Contestation to Authority; TR = Traditionalism; SA = Submission to Authority; Political self-categorization was coded as 1 = Left; 2 = Center-Left; 3 = Center; 4 = Center-Right; 5 = Right.

**Supplementary Material C - Fit indices of CFAs using Samples 2, 3 and 4a merged**

In this Supplementary Material C, we sought to replicate the dimensional structures assessed in the Main Document with Samples 2, 3 and 4a merged (*N*_total_ = 1,300). A CFA using the WLSMV estimation method was conducted. Not only the single-order structures tested in the Main Document were assessed, but also second-order structures because Bizumic and Duckitt (2018) indicated that a 1*3 higher-order solution had the best fit indices across all models they tested.

In our Sample, we tested a 1*3 higher-order solution as indicated by Bizumic and Duckitt (2018) and a 1*4 higher-order solution, since the four-dimensional model was the one with best fit indices in our Samples. As shown in the Table S2 below, the single-order four-dimensional structure was still the only adequate (X²/df = 3.64; CFI = .98; TLI = .97; RMSEA = .05), as indicated in the Main Document. The 1*3 higher-order solution did not present adequate fit indices (X²/df = 54.39; CFI = .44; TLI = .30; RMSEA = .20) and neither did the 1*4 higher-order solution (X²/df = 51.95; CFI = .47; TLI = .34; RMSEA = .20). Similarly, the single-order one-dimensional structure (X²/df = 37.33; CFI = .61; TLI = .53; RMSEA = .17) and the single-order three-dimensional structure (X²/df = 21.13; CFI = .80; TLI = .74; RMSEA = .13) also presented inadequate fit to the data. Hence, the results reported in the Main Document were replicated. Future studies should thus try to address the reasons why the higher-order dimensions were not adequate in our Brazilian samples whereas presenting the best fit to Bizumic and Duckitt (2018) data.

**Table S2.** *Model Fit for Single-Order Four-Factor, Single-Order Three-Factor, Single-Order One-Factor, and Higher-Order 1*3 and 1*4 Dimensions*

| Model | χ²/*df* | CFI | TLI | RMSEA | χ²_difference_ test | | |
| --- | --- | --- | --- | --- | --- | --- | --- |
|  |  |  |  |  | χ² _difference_ | *df* _difference_ | *P* |
| Four-Factor (AT, TR, SA, CA) | 3.64 | .98 | .97 | .05 | - | - | - |
| Three-Factor proposed by Duckitt et al. (2010; AT, TR, SA+CA) | 21.13 | .80 | .74 | .13 | 89.96 | 3 | < .001 |
| One-Factor Proposed by Altemeyer (1981) | 37.33 | .61 | .53 | .17 | -1864.57 | 1 | 1 |
| Higher-order Four-Factor (1*4 – AT, TR, SA, CA * Higher Order) | 51.95 | .47 | .34 | .20 | 715.84 | 2 | < .001 |
| Higher-order Three-Factor (1*3 – AT, TR, SA+CA * Higher Order) | 54.39 | .44 | .30 | .20 | -940.16 | 0 | - |

Note: AT = Authoritarianism; CA = Contestation to Authority; TR = Traditionalism; SA = Submission to Authority
